# BCD-WERT: a novel approach for breast cancer detection using whale optimization based efficient features and extremely randomized tree algorithm

**DOI:** 10.7717/peerj-cs.390

**Published:** 2021-03-12

**Authors:** Shafaq Abbas, Zunera Jalil, Abdul Rehman Javed, Iqra Batool, Mohammad Zubair Khan, Abdulfattah Noorwali, Thippa Reddy Gadekallu, Aqsa Akbar

**Affiliations:** 1 Department of Computer Science, Air University, Islamabad, Pakistan; 2 Department of Cyber Security, Air University, Islamabad, Pakistan; 3 Department of Computer Science, College of Computer Science and Engineering, Taibah University, Madinah, Saudi Arabia; 4 Electrical Engineering Department, Umm Al-Qura University, Makkah, Saudi Arabia; 5 School of Information Technology and Engineering, Vellore Institute of Technology University, Tamil Nadu, India

**Keywords:** Breast cancer, Machine learning, Whale optimization algorithm, Support vector machine

## Abstract

Breast cancer is one of the leading causes of death in the current age. It often results in subpar living conditions for a patient as they have to go through expensive and painful treatments to fight this cancer. One in eight women all over the world is affected by this disease. Almost half a million women annually do not survive this fight and die from this disease. Machine learning algorithms have proven to outperform all existing solutions for the prediction of breast cancer using models built on the previously available data. In this paper, a novel approach named BCD-WERT is proposed that utilizes the Extremely Randomized Tree and Whale Optimization Algorithm (WOA) for efficient feature selection and classification. WOA reduces the dimensionality of the dataset and extracts the relevant features for accurate classification. Experimental results on state-of-the-art comprehensive dataset demonstrated improved performance in comparison with eight other machine learning algorithms: Support Vector Machine (SVM), Random Forest, Kernel Support Vector Machine, Decision Tree, Logistic Regression, Stochastic Gradient Descent, Gaussian Naive Bayes and k-Nearest Neighbor. BCD-WERT outperformed all with the highest accuracy rate of 99.30% followed by SVM achieving 98.60% accuracy. Experimental results also reveal the effectiveness of feature selection techniques in improving prediction accuracy.

## Introduction

Breast cancer is a life-threatening disease that affects the lives of many women. It is also the second leading cause of deaths occurring due to cancer among females (Meera & Nalini, 2018; Kamel, YaghoubZadeh & Kheirabadi, 2019). Breast cancer annually affects one million people and results in over 400,000 deaths globally (Meera & Nalini, 2018). It is a benign or malignant tumor, caused by uncontrolled growth and division of cells inside the breast (Kamel, YaghoubZadeh & Kheirabadi, 2019). The non-cancerous tumors are called benign which are not life-threatening and can be treated with medicine. The cancerous tumors are known as malignant and if left untreated, the patient can die. These tumors appear as a lump in the breast and can be diagnosed using X-rays. For early detection, the patient must immediately consult with their healthcare provider if such a lump appears in their breast (Meera & Nalini, 2018; Alghunaim & Al-Baity, 2019).

Family history, age, genes, dietary habits, and lifestyle are some of the leading factors causing this disease (Kamel, YaghoubZadeh & Kheirabadi, 2019). Treatments costs are elevated for breast cancer and also, there are severe side effects after long-term usage of medications by patients. Many causalities occur due to the detection of cancer at a stage where it becomes impossible to cure this disease (Kamel, YaghoubZadeh & Kheirabadi, 2019; Alghunaim & Al-Baity, 2019). According to medical professionals, early detection of disease is crucial. Recently, medical practitioners have developed a greater interest in the prediction and detection of breast cancer using machine learning algorithms. Machine learning is a subset of Artificial Intelligence that provides the ability to learn and improve from past experiences without being explicitly programmed (Khourdifi & Bahaj, 2018). In medical discipline, vast and rich information is available about several diseases such as cancer, diabetes, COVID (Sahlol et al., 2020a, 2020b; Dev et al., 2020; Zehra et al., 2020; Khamparia et al., 2020; Gupta, Chakraborty & Gupta, 2019; Chakraborty et al., 2016; Chakraborty, Gupta & Ghosh, 2015). The patterns hidden inside this data can be used to dig in meaningful relationships (Jhaveri & Patel, 2017). Machine learning algorithms are already being incorporated in improving the health, protection and welfare of billions of people (Javed et al., 2020a, 2020b, 2021; Shabbir et al., 2021; Sarwar & Javed, 2019). Many machine learning algorithms such as DT, SVM, RF, Naive Bayes and KNN are being used in the medical field and have provided remarkable results in early-stage disease prediction and diagnosis (Karim et al., 2020; Ak, 2020; Usman Sarwar et al., 2020; Rehman et al., 2020; Javed et al., 2020c).

The virtues of machine learning algorithms are remarkable but there is room for improvement in certain domains such as prediction and detection accuracy, computational complexity, and execution time while using volumes of data (Sagar, Jhaveri & Borrego, 2020; Desai & Jhaveri, 2018). Performance can be improved by careful evaluation and a combination of several optimization techniques available (Kamel, YaghoubZadeh & Kheirabadi, 2019). The performance of these classification algorithms can be improved by using feature selection algorithms such as Grey Wolf Optimization (GWO), Particle Swarm Optimization (PSO) and WOA (Vieira et al., 2013). As the data stored in medical systems is vast and complex, it often contains unnecessary features that may mislead the classification algorithm and negatively affect the results (Chakraborty et al., 2016). Similarly, the relevant features can exclusively optimize the algorithm and correctly predict the class. It is of paramount importance to distinguish relevant features from irrelevant ones and utilize relevant for accurate prediction. Feature selection is used to eliminate the unnecessary data from the dataset and helps improve the accuracy rate of the prediction algorithm by reducing the dimensionality of the data (Kamel, YaghoubZadeh & Kheirabadi, 2019; Alghunaim & Al-Baity, 2019).

In this article, a novel hybrid classification approach named *BCD-WERT* is adopted that utilizes feature selection and machine learning techniques for breast cancer prediction. *BCD-WERT* consists of WOA to reduce the high dimensionality of the data and classification is performed using Extremely Randomized Tree (ERT) algorithm. The selected features are given as input to other classification algorithms for breast cancer prediction. These algorithms include SVM, KNN, KSVM, GNB, SGD, DT, LR and RF. *BCD-WERT* achieves promising classification performance using WOA and ERT in comparison with other classifier and state-of-the-art studies.

The rest of the article is structured as follows: “Literature Review” provides a brief literature review of the several feature selection and data mining techniques used for prediction. In “BCD-WERT Approach”, our proposed hybrid approach is discussed in detail. A brief introduction to the WOA and classifiers is discussed. The experimental results of the proposed approach are provided in “Experimental Analysis and Results”. Discussion of results is presented in the “Discussion” section. “Conclusion and Future Work” presents the conclusion of this research study and highlights dimensions of future research.

## Literature review

Breast cancer takes the lives of almost half a million women every year (Meera & Nalini, 2018). It should be detected and cured as early as possible. Several predictions and medical treatment options are adopted by doctors to combat this fatal disease. Early prediction and detection can save many lives. Machine learning algorithms are also being used to predict the disease and have shown promising results in most of the cases. The algorithms proposed so far can be placed in two categories: (a) feature selection based methods and (b) data mining-based methods. The detailed review is as follows:

### Feature selection based methods

Ala’M et al. (2018) proposed a hybrid technique based on SVM and WOA. The model was developed for spam detection and provides insight into which features play a deciding role in the detection of spam. The proposed model was evaluated on several datasets in a different context (i.e., Arabic, English, Spanish and Korean). It achieved 99% accuracy on Arabic language datasets, 91% on the Spanish language, 96% on the English language, and 95% on the Korean language dataset. Kamel, YaghoubZadeh & Kheirabadi (2019) utilized a combination of GWO and SVM for breast cancer prediction. UCI dataset was used for experiments. It was found that SVM-GWO achieves 100% accuracy for prediction whereas a 99.29% accuracy rate was achieved without feature selection. The results were compared with other classification algorithms and it was found that in terms of sensitivity, accuracy, and specificity, SVM-GWO outperforms all. As compared to work done in the literature, the proposed method increased diagnosis accuracy by 27.68%. Sayed et al. (2016) proposed a classification technique based on a WOA and Support Vector Machine (SVM) for the prediction of breast cancer. In this model, the kNN classifier was used to extract a subset of features with the best fitness value. WOA was used for the selection of optimal features among the obtained subset. Finally, these best features were fed to SVM classifier for classification of instances. This model achieved an accuracy of 98.77%. Besides this, Mohammed, Umar & Rashid (2019) proposed a meta-heuristic algorithm named “Feature Selection based on WOA (FSWOA)”. FSWOA was used to reduce the dimensionality of medical data. The accuracy of the proposed FSWOA observed on several medical datasets was 87.10% for Hepatitis, 97.86% for Breast Cancer, 78.57% for Pima Indians Diabetes and 77.05% for Starlog Disease.

Sakri, Rashid & Zain (2018) compared the results of several algorithms for predicting breast cancer recurrence. This study used the PSO algorithm for feature selection in combination with renowned classifiers fast DT learner, KNN, and NB classifier. Experimental results showed that without PSO based feature selection, the highest accuracy achieved was 76.3%. With PSO feature selection, NB provided the highest accuracy that was 81.3%. Sharifi & Alizadeh (2019) used EM to analyze the data and after normalizing, the neural network multilayer perceptron structure with WOA was used to predict breast cancer. The accuracy achieved after performing preprocessing and reducing dimensions of the dataset was 99% and it comes out to be a good machine learning method in comparison to other techniques used. Asri et al. (2016) discussed the performance of four classifiers C4.5, SVM, NB and KNN to predict breast cancer. The main purpose of the study was to evaluate the performance of algorithms based on accuracy, sensitivity, and specificity. SVM gave the highest accuracy of 97% according to the experimental results on the Wisconsin dataset. Authors in Sivakami & Saraswathi (2015) compared classifications techniques for breast cancer prediction. NB, SMO, Instance-based learning (IBL) and DTSVM. This work proposed a hybrid methodology to predict the fatal disease and to alarm the consequences of the disease. Two methods were used to predict the status of the disease, option extraction and information treatment.

Dubey, Gupta & Jain (2016) used the *K*-means algorithm to predict breast cancer at early stages, and used Breast Cancer Wisconsin dataset. Centroids and distance measures were used to compute the results. Positive prediction accuracy of 92% was achieved. Different correlation techniques were used and, Manhattan and Euclidean proved to be more effective than Pearson correlation, and it was observed that *K* mean algorithm can be effectively used for classification.

### Data mining based methods

Meera & Nalini (2018) conducted a comparative analysis of data mining methods for breast cancer prediction based on execution time and classification accuracy as performance measures. This study used NB and J48 data mining algorithms. The performance of NB and J48 was compared in terms of classification accuracy and execution time. The results show that NB had an accuracy of 64% whereas J48 had an accuracy of 60%. This study concluded that NB is a better classification algorithm with a higher accuracy rate and less execution time as compared to J48. Khourdifi & Bahaj (2018) conducted a comparative analysis of KNN, SVM, RF and NB for cancer detection accuracy. The result showed that SVM produced the highest accuracy of 97.9%.

Alghunaim & Al-Baity (2019) conducted a comprehensive comparative study exploring how well machine learning algorithms perform in breast cancer prediction using big data in terms of performance, effectiveness, and efficiency. Both Spark and WEKA platforms were used for experiments as these facilitate scalable and non-scalable environments respectively. The performance of SVM, RF and DTs were compared on DNA Methylation (DM), Gene Expression (GE), and mixed combined datasets. The results show that SVM outperformed DT and RF with all three datasets with 99.68%, 98.73%, and 97.33% accuracy respectively. This study found that the GE dataset is the best choice among the three datasets to accurately predict breast cancer. Al-Zoubi et al. (2019) conducted experiments to compare the performance of NB, C4.5, SVM and KNN. Experimental results showed that SVM is the best classifier and gave the highest prediction accuracy of 96.99% (Al-Zoubi et al., 2019). Several researchers have explored this problem in past. Table 1 provides the comparison of the previous studies, the solutions proposed, and associated limitations.

**Table 1:** Literature review summary.

Authors	Problem solved	Limitations
Alghunaim & Al-Baity (2019)	Cancer Prediction by using SVM, RF and DT	RF does not gives precise prediction values
Kamel, YaghoubZadeh & Kheirabadi (2019)	Cancer prediction by using combination of SVM and GWO	Grey Wolf Optimization algorithm has bad local searching ability
Salama, Abdelhalim & Zeid (2012)	MLP and J48 classifiers with WBC dataset	MLP is fully connected and includes many parameters
Salama, Abdelhalim & Zeid (2012)	SMO and MLP for breast cancer prediction by using the WDBC dataset	Performance of SMO with SVM is not good on noisy data
Dubey, Gupta & Jain (2016)	K-means algorithm was used to predict breast cancer on BCW dataset	Using K-mean with foggy clusters may cause difference in final clusters
Sivakami & Saraswathi (2015)	Comparison of three classifications for breast cancer prediction	Degraded performance with increased number of features
Asri et al. (2016)	SVM, NB, KNN, and C4.5 on WBC dataset for breast cancer	C4. 5 classifier with SVM faces overfitting
Karim et al. (2019)	NB and J48 data mining algorithms for breast cancer prediction	Feature selection can lead to cost effectiveness but J48 and NB in this study have not used selected feature

## *BCD-WERT* Approach

**Figure 1:**
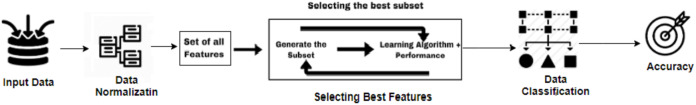
Proposed *BCD-WERT* approach.

In this study, a classification based method named *BCD-WERT* is proposed for breast cancer prediction. *BCD-WERT* uses WOA based efficient features and well-known classification algorithms. *BCD-WERT* consists of four main phases. In phase I, the dataset is collected for experiments. The Wisconsin breast cancer diagnostic dataset from Kaggle online dataset library is used in this study. In phase II, data is preprocessed and cleaned accurately for prediction. In phase III, the WOA is used for feature selection. In phase IV, selected features are used for the training of the classification models. Several classification methods are used for the classification of testing data. For this study, the WOA and all other classification models are implemented using Python. Figure 1 presents the working of the proposed approach and a detailed Algorithm 1 is presented below.

**Algorithm 1:** Proposed solution algorithm.

**Input:** *Reading* ← *DatasetReadings* **Output:** Benign, Malign **Evaluation Measures:** Accuracy, F-Score, Recall, Precision 1: *i* ← [Reading] {Current Instance} 2: *T* ← [ ] {Total Instances} 3: *P* ← [ ] {Predicted Confidence} 4: *C* ← [ ] {Targeted Confidence} 5: *L* ← [Benign,Malign] { Target Class Labels} 6: **Find Best Feature by usingWOA** 7: Initialize the population Yj(1,2,3,…,n) 8: Initialize x, P and z 9: Calculate the best feature fitness for each search value 10: Y= the best Search value 11: **Function WOA** (population,x,P,z,Max_iter) 12: i=1 13: **while** i ≤ Max iter **do** 14: **for** Each Search Value **do** 15: **if** P ≥ 1 **then** 16: Update the position of current search value 17: **else if** P ≥ 1 **then** 18: Choose random search value Y 19: Update the position of current Value. 20: **end if** 21: **end for** 22: Update x,P,z 23: Update Yj if Y got better Solution 24: I=i+1 25: **end while** 26: Return Yj 27: **End Function** 28: **Function Classifiers**(Feature Selected) 29: Extract Best feature from dataset 30: Do Computation on selected feature 31: **while** loop until margin constraints violating points **do** 32: K splits {sp1,…spk} sp(i) is a random split 33: **Return** split sp* score(sp*) 34: **end while** 35: **return yi** 36: Compute Accuracy and Confusion Matrix

### Data acquisition

The dataset used in this study is Wisconsin Breast Cancer Diagnostic dataset (WBCD) that is acquired from the online Kaggle dataset library (University of Wisconsin, 2016). It contains 569 instances and has 30 attributes. There are two classes an instance can belong to, Malignant and Benign. The cancerous class is represented by 1 and non-cancerous is represented by 0 in the dataset. Cancer can be Malignant when cell growth is uncontrollable. The motivation behind using this dataset is to propose an approach to predict malignant breast cancer. The spreading and growth of the cancerous cells can become life-threatening. Malignant tumors rapidly grow and can quickly spread to other body parts.

### Data pre-processing

After the data is acquired, pre-processing (Reddy et al., 2020) is performed on the selected dataset. Data normalization is used to prepare data for classification and the next step is to clean the data, by removing irrelevant and incomplete records. This is done to make sure the data is consistent and the dataset contains no missing values. After data normalization, random splitting of the dataset is done into two subsets (i.e., training data and testing data). Data is preprocessed to avoid over-fitting of the proposed model.

### Feature selection

Whale Optimization Algorithm (WOA) is used to perform feature selection. WOA is used to find the best features among 30 attributes to feed the classification models. WOA is a meta-heuristic optimization proposed by Mirjalili & Lewis (2016). It is a nature-inspired approach that mimics the real-life behavior of a group of the largest mammals on the planet. WOA is a swarm-based technique that is designed based on the social behavior of humpback whales and takes inspiration from the bubble-net strategy unique to them for hunting in the ocean (Gadekallu et al., 2020; Iwendi et al., 2020). Humpback whales are the largest group of baleen whales and they usually spend their days as a group. They hunt small groups of krill and small fishes close to the surface by creating bubbles along a spiral path around their prey and then they swim up to the surface following this path (Mirjalili & Lewis, 2016; Karim et al., 2020).

Using this bubble-net hunting mechanism, Mirjalili & Lewis (2016) proposed a mathematical model and algorithm to solve optimization problems (De Lima, De Oliveira Roque e Lima & Barbosa, 2020). The WOA consists of three main steps as discussed down below:

- **Encircling Prey:** To hunt, humpback whales can identify the location of their prey and attack them by encircling them. The WOA also works on a similar principal. As the best solution is not known beforehand, WOA assumes that the target prey is the current best solution found in the current iteration and that it is closest to the optimum solution. Once the current best solution or agent is found, other search agents also update their positions relevant to the selected solution encircling the prey solution. Eqs. (1)–(4) represent this behavior:

(1) }{}D = |CX(t) - X(t)|

(2) }{}X(t + 1) = X(t) - AD

(3) }{}A = 2ar - a

(4) }{}C = 2r

Here *t* = the current iteration, *C* = coefficient vectors, X* = position vector of best solution which is updated if better solution is found, *X* = position vector, *a* = value between 2 and 0, and *r* = random vector (De Lima, De Oliveira Roque e Lima & Barbosa, 2020).

- **Bubble-Net Attack:** This is the exploitation stage where humpback whales simultaneously move towards their prey in a shrinking circle and move in the spiral path simultaneously. WOA assumes that there is a 50% probability of a whale to select either of the following methods to catch their prey.
- **Search For Prey:** In addition to the bubble-net attack, the humpback whale also randomly searches for their prey. This is the exploration stage and the whale hunt the prey relevant to the position of other whales. To mimic this behavior, the value of ‘A’ vector is kept either less than −1 or greater than 1 to help WOA conduct a global search. This behavior is expressed in the form of the Eqs. (5) and (6):

(5) }{}D = |CXrandX|

(6) }{}X(t + 1) = Xrand - AD

Here, Xrand is a random position vector chosen from the current population Karim et al. (2020).

### Classification models

Several machine learning algorithms (i.e., ERT, DT, KNN, SGD, RF, LR, KSVM, GNB and SVM) are used for the classification of the WBDC dataset. The basic purpose of using these classifiers is to predict the class label for a given instance. Details about the classifiers are discussed below:

- Extremely Randomized Tree (ERT) algorithm, an ensemble of several decision trees is used for classification. This represents a forest of decision trees which is similar to the RF algorithm but differs in the way the DT is built. To split each node in the DT, every DT selects the best feature based on some selected criteria from a list of randomly selected K attributes. The Extra Trees algorithm creates unpruned trees and a large number of decision trees using the training dataset. For regression and majority voting, averaging the predictions is done by this algorithm to make final predictions of all decision trees (Karim et al., 2019).
- Support Vector Machine (SVM) is trained using training data for each class, it can categorize new samples (Ak, 2020). It is a non-probabilistic binary linear classifier (Karim et al., 2019). SVM utilizes the right hyper-plane to differentiate between two classes (Karim et al., 2019). Researchers have proposed many applications of SVM including attack detection and cancer diagnosis (Vieira et al., 2013).
- K-Nearest Neighbor (kNN) is an instance-based algorithm that classifies a sample based on the classes of its nearest neighbor. In this algorithm, K’s closest examples are selected from the neighborhood of the sample that needs to be classified. A vote is taken among these examples and the new sample is assigned the most occurring class among its neighbors. A distance measure such as Euclidean distance is used to find the nearest neighbors of the sample (Latchoumi & Parthiban, 2017).
- Naive Bayes (NB) classifies samples using a probabilistic classification approach. NB is considered a simple and popular algorithm for many classification problems. GNB is a variant of the NB algorithm which is used for the classification of continuous data using the gaussian distribution. In GNB, the standard deviation and mean of each given class corresponding to every sample in the training data is calculated and classification is performed according to these calculations (Kamel, Abdulah & Al-Tuwaijari, 2019).
- Stochastic Gradient Descent (SGD) is an optimization technique for training a classification model. Unlike gradient descent which utilizes all data samples to calculate the gradient of the cost function, SGD uses one randomly selected sample in each iteration. Although more noise is introduced in SGD due to the random selection of samples. It is still considered as much faster than GD to reach minima (Vieira et al., 2013).
- Random Forest (RF) technique is an ensemble of tree-structured learning classifiers. It classifies a new sample based on the most occurring prediction made by these algorithms. Feature selection is used to grow the trees and at each node, random features are selected for splitting. This helps in minimizing over-fitting and as a result, RF classification is fast (Akar & Gungor, 2012).
- Logistic Regression (LR), also known as parametric classification, utilizes “maximum likelihood estimation” for classification. It performs a probability analysis of the entire data to assign classes to new samples (Dreiseitl & Ohno-Machado, 2002).
- Decision Tree (DT) algorithm derive rules from the given training dataset and build a tree-like structure. The tree is grown by splitting nodes on the values of a feature. A criterion, such as information gain, is used to select the feature that best splits the tree and leads to a maximum decrease in entropy (Dreiseitl & Ohno-Machado, 2002).
- Kernel Support Vector Machine (KSVM) uses different kernel functions for the decision function. A linear SVM is turned into a non-linear model by applying the kernel trick to the model; replacing predictors with a kernel function.

## Experimental analysis and results

For experimentation, the Wisconsin breast cancer dataset (Asri et al., 2016) is used for the detection of breast cancer. The dataset is split into 75% for training and 25% for testing. To analyze the performance of the proposed classification model, the performance measures such as accuracy, f-score, recall, and precision are used. The best feature is predicted using WOA and then classification models perform prediction. The proposed model reduces the number of features and is capable of handling large data for cancer prediction with better accuracy. *BCD-WERT* achieves the highest accuracy of 99.30% with Extremely Randomized Tree (ERT) classifier. The performance of other classifiers can be observed in Table 2.

**Figure 2:**
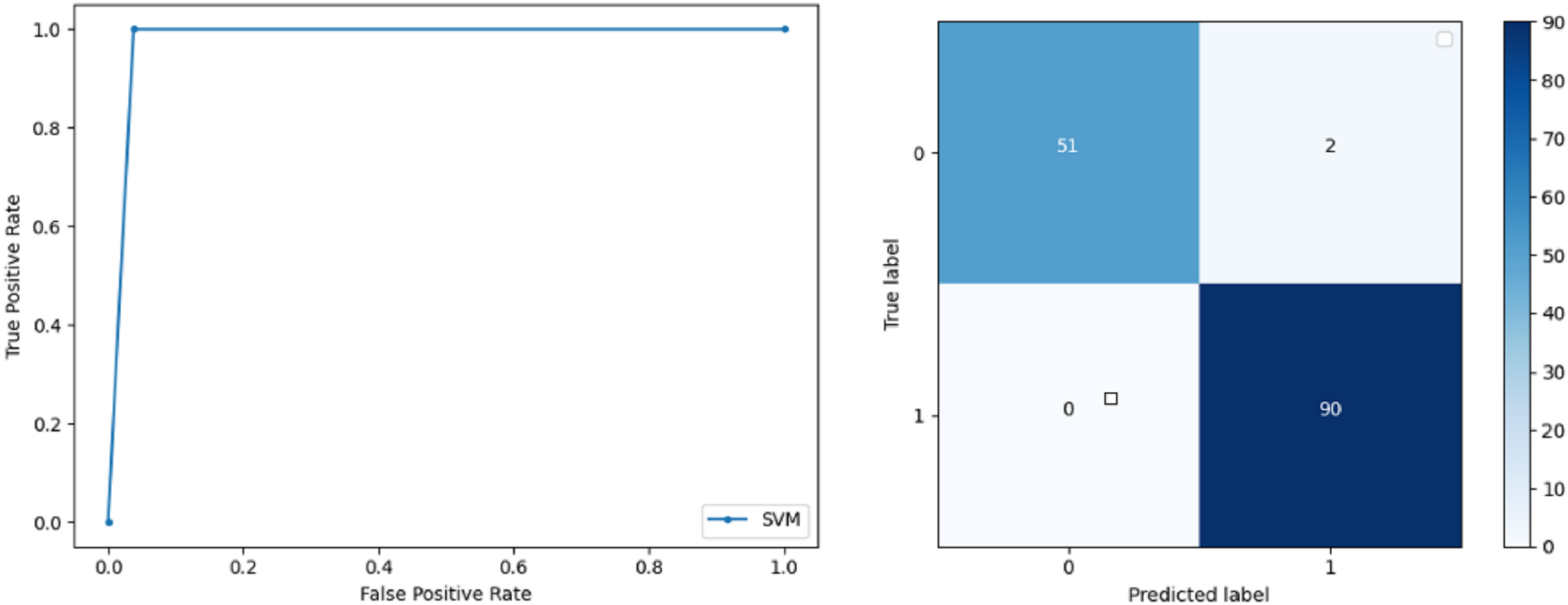
BCD with support vector machine classifier: ROC curve and confusion matrix.

**Figure 3:**
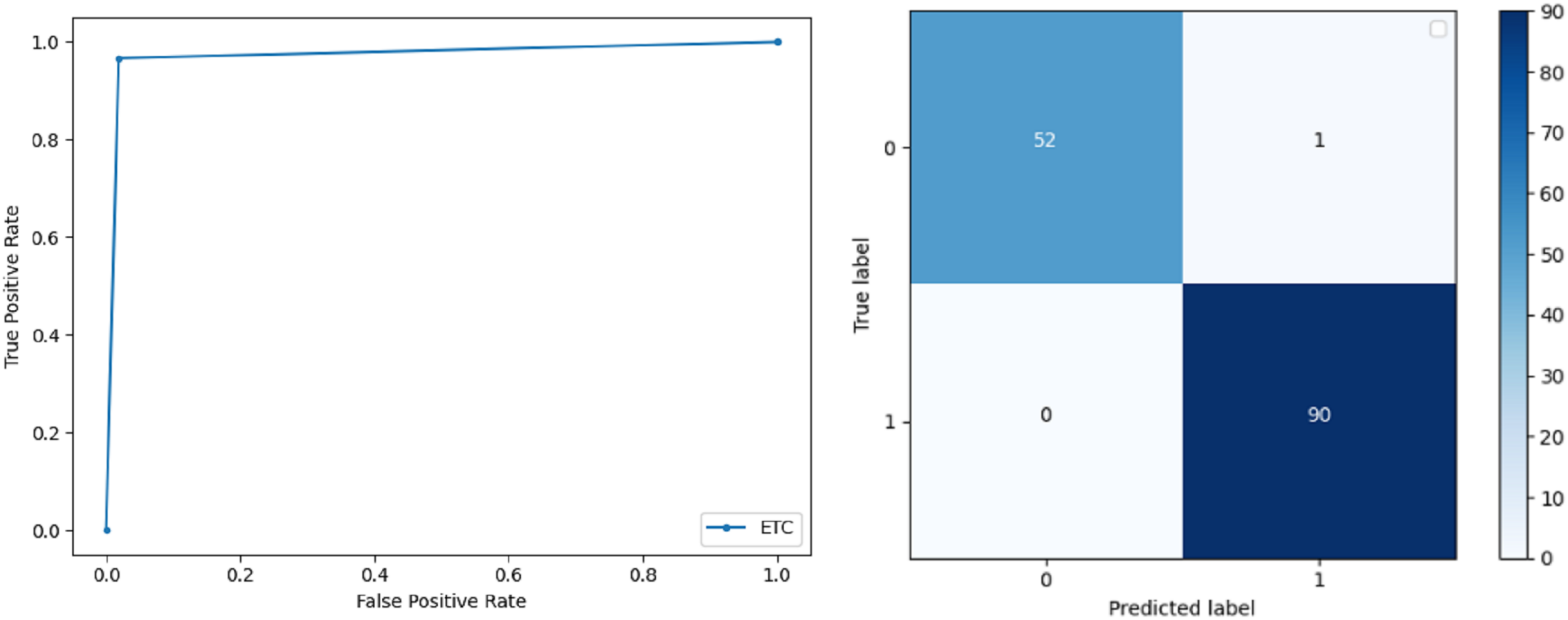
BCD with extra tree classifier: ROC curve and confusion matrix.

**Figure 4:**
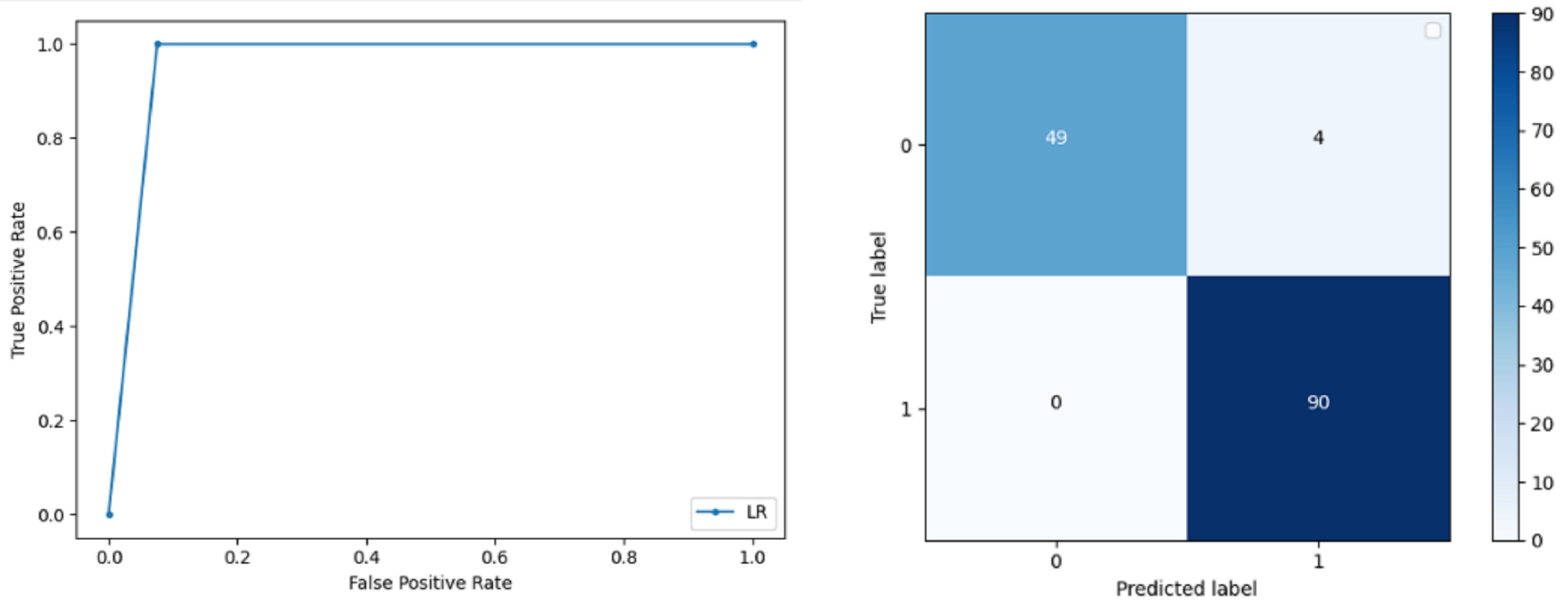
BCD with logistic regression classifier: ROC curve and confusion matrix.

**Figure 5:**
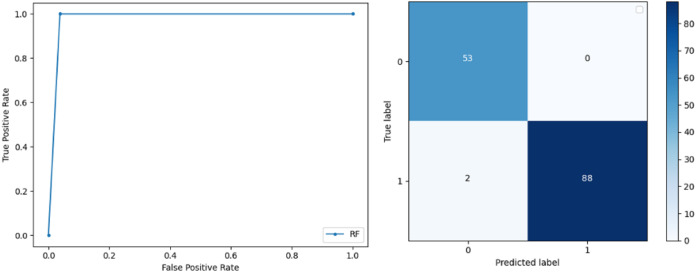
BCD with random forest classifier: ROC curve and confusion matrix.

**Figure 6:**
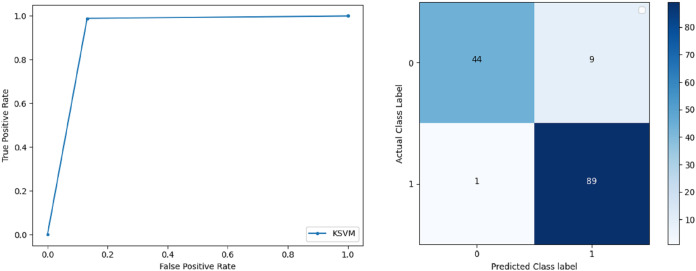
BCD with Kernel support vector machine classifier: ROC curve and confusion matrix.

**Figure 7:**
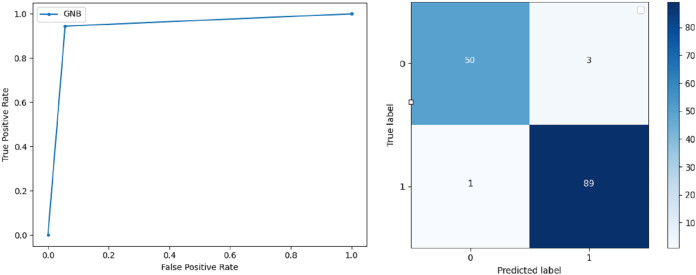
BCD with Gaussian Naive Bayes classifier: ROC curve and confusion matrix.

**Figure 8:**
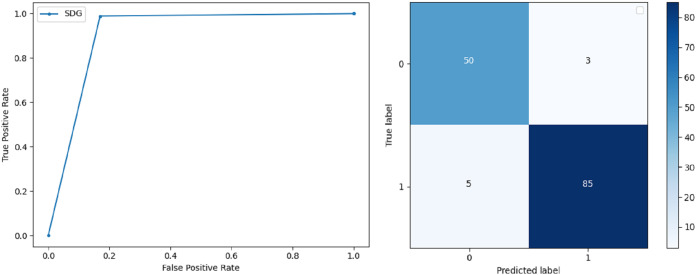
BCD with stochastic Gaussian Descent classifier: ROC curve and confusion matrix.

**Figure 9:**
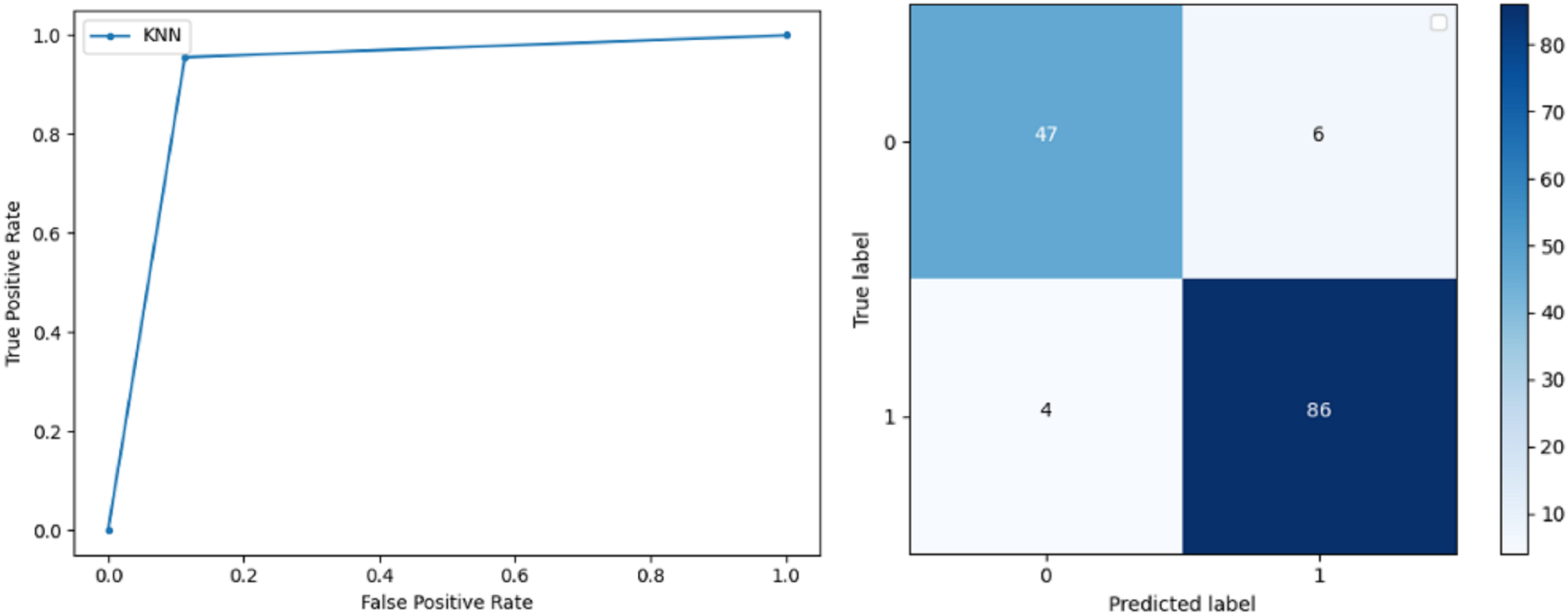
BCD with K-nearest neighbour classifier: ROC curve and confusion matrix.

**Figure 10:**
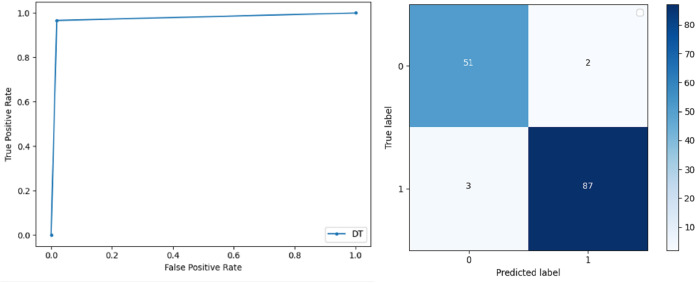
BCD with decision tree classifier: ROC curve and confusion matrix.

- **Support Vector Machine:** SVM classified the data with 98.60% accuracy making use of selected features extracted through WOA. The precision of both classes 0 and 1 is 1.00 and 0.98 respectively. Precision shows that the number of correctly identified positive results in both classes is more than the incorrect ones. F-score of the classification model for 0 and 1 class in the SVM case is 0.98 and 0.99 respectively and the recall for both classes is 0.96 and 1.00 respectively. These results depict that SVM can be used to predict breast cancer and early diagnosis is possible for most cases. Receiver Operating Characteristics (ROC) curve and confusion matrix of SVM classification is presented in Fig. 2.
- **Extremely Randomized Tree:** ERT outperforms all other classifiers used in our experiments and achieves an accuracy of 99.30%. This is achieved since this classifier aggregates results of multiple co-related DTs and uses a random sample for this purpose. The accuracy achieved shows that this classifier can be effectively used to predict this fatal disease. The f-score of both 0 and 1 class, in this case, is 1.00 and 0.99 and the recall for 0 and 1 is 0.98 and 1.00 respectively. Precision shows that correctly identified positive results are enough and the value for both classes is 0.99. ROC curve and confusion matrix of ERT classification is presented in Fig. 3.
- **Logistic Regression:** LR classification is used to estimate the perimeters of the logistic model and accuracy achieved using LR classifier is 97.01%. The precision measure of regression for 0 and 1 class is 1.00 and 0.96 respectively and the f-score achieved is 0.96 and 0.98 respectively. The recall of the classifier is 0.98 and 0.96 respectively. ROC curve and confusion matrix of LR classifier can be seen in Fig. 4.
- **Random Forest:** RF gives high accuracy with large datasets. The accuracy after applying this classifier on the Wisconsin breast cancer dataset is 98.60% and the f-score calculated for 0 and 1 class is 0.98 and 0.99 respectively. The precision measure for RF is 0.96 and 1.00 for 0 and 1 class respectively. The recall of this classifier for classes 0 and 1 is 1.00 and 0.98 respectively. These results show that RF performs well on a large dataset. ROC curve and confusion matrix of RF classification is presented in Fig. 5.
- **Kernel Support Vector Machine:** KSVM operates in high dimensions and performs pattern analysis. The accuracy achieved by KSVM in the proposed solution is 94.41% and precision for class 0 and 1 is 0.98 and 0.93 respectively. The F-score for KSVM in both 0 and 1 class is 0.92 and 0.96 respectively. Recall for the KSVM classifier obtained is 0.87 and 0.99 for both 0 and 1 class respectively. ROC curve and confusion matrix of KSVM classification is presented in Fig. 6.
- **Gaussian Naive Bayes:** GNB is used to find strong independence between the features of the dataset and it performs well when applied to the selected features of a huge dataset. The accuracy achieved by the classifier is 94.40% and the precision measurement of the 0 and 1 class is 0.91 and 0.97 respectively. The F-score achieved by using GNB is 0.93 and 0.96 for both 0 and 1 class respectively. These results depict that this classifier can be used for the prediction of cancer but other classifiers outperformed GNB in terms of accuracy. ROC curve and confusion matrix of GNB classification is presented in Fig. 7.
- **Stochastic Gradient Descent:** SGD performs well on large scale dataset but it is sensitive when it comes to feature scaling. The accuracy achieved by the classifier is 93.00% and the F-score for 0 and 1 class is 0.90 and 0.95 respectively. The precision and recall achieved for 0 and 1 class are 0.98, 0.91 and 0.83, 0.99 respectively. ROC curve and confusion matrix of SGD based classification can be observed in Fig. 8.
- **K-Nearest Neighbour:** KNN relies on distances for classification and training and here data normalization can improve accuracy. The accuracy achieved by the KNN used in the proposed solution is 93.20%. F-score and precision of class 0 and 1 is 0.90, 0.95 and 0.92, 0.93 respectively.The results obtained by using K-Nearest Neighbour are adequate when there is no other classifier but it performed worse than the other classifiers used because of the value of the k selected on a random basis. The recall of the proposed solution is 0.89 and 0.96 for classes 0 and 1 respectively. ROC curve and confusion matrix of kNN classifier is presented in Fig. 9.
- **Decision Trees:** DT is used to split data according to a certain parameter. The accuracy achieved by this classifier is 97.20% and the *F*-score and precision of class 0 and 1 are 0.96, 0.98 and 0.95 and 0.99 respectively. The recall obtained is 0.98 and 0.97 for 0 and 1 class respectively. ROC curve and conclusion matrix of DT based classification is presented in Fig. 10.

**Table 2:** Comparison with machine learning algorithm.

Classification model	Accuracy (%)	Precision	*F*-score	Recall
K-nearest neighbour	93.02	0.92	0.9	0.89
		0.93	0.95	0.96
Decision tree	97.20	0.95	0.96	0.98
		0.99	0.98	0.97
Stochastic gradient descent	93.00	0.98	0.90	0.83
		0.91	0.95	0.99
Kernel support vector machine	94.41	0.98	0.92	0.87
		0.93	0.96	0.99
Gaussian naive bayes	94.40	0.91	0.93	0.94
		0.97	0.96	0.94
Random forest	98.60	0.96	0.98	1.00
		1.00	0.99	0.98
Logistic regression	97.01	1.00	0.96	0.92
		0.96	0.98	0.98
Support vector machine	98.60	1.00	0.98	0.96
		0.98	0.99	1.00
Extra tree	99.30	1.00	0.99	0.98
		0.99	0.99	1.00

## Discussion

There exist several randomization methods for trees and classification but building completely random trees are still far away. Extra Randomized Tree algorithm works solely on random behavior and selects the cut points for the tree. In the end, a randomized tree is built and then prediction and classification are done using that tree. The proposed approach is capable of accurately predicting whether a patient has breast cancer or not, even when limited data is available as it only chooses the best feature for classification. Medical practitioners can easily use this model to check if a patient is at risk of getting breast cancer. Nowadays, medical facilities are getting expensive so this model can help in the early diagnosis of cancer in patients (Sivakami & Saraswathi, 2015). Table 2 shows the results obtained from the proposed approach and the ERT classifier outperforms all other classifiers used for breast cancer prediction. The accuracy achieved through SVM, ERT, DT, LR and RF is higher as compared to other algorithms used in this study. These can be used to classify and predict breast cancer to avoid the spreading of cancerous cells in the body. If the disease is diagnosed timely, many patients can be saved from facing dire conditions in later stages and the treatment of this disease can be started on time. All classifiers used in this study performed well except SGD and KNN. The reason behind this is because the KNN classifier works on distance measures, the value of k also has an immense effect on the accuracy of the model. SDG performance lacks because of the usage of the same learning rate for all parameters. All the other classifiers were able to classify the dataset with a high accuracy rate. ROC and confusion matrix of each classifier shows the performance of the classifier and ERT proves to be the best classifier among all nine classifiers used in this study. Table 3 shows the result summary and comparison with the existing solutions. The ROC curve and confusion matrix of each classifier is given.

**Table 3:** Comparison with state-of-the-art studies.

Authors	Dataset used	Algorithm	Results
Chaurasia & Pal (2017)	Breast cancer medical dataset	Sequential minimal optimization, K-nearest neighbor, decision tree	Best results found by SMO, Accuracy: 96.19%
Zheng, Yoon & Lam (2013)	Breast cancer medical dataset	K-mean and support vector machine	Accuracy: 97.38%
Alghunaim & Al-Baity (2019)	Gene expression	Support vector machine, RF, and decision trees	Support vector machine without feature selection outperformed with an accuracy of 99.68%,
Salama, Abdelhalim & Zeid (2012)	Wisconsin breast cancer dataset	Support vector machine	SVM accuracy: 96.99%
Proposed Solution (BCD-WERT)	Wisconsin breast cancer dataset	SVM, KNN, RF, LR, DT, GNB, SGD, ERT, KSVM	ERT outperformed other classifiers and achieved an accuracy of 99.30%

## Conclusion and future work

In this paper, a novel approach named *BCD-WERT* is proposed that utilizes WOA and Extra Randomized Tree (ERT) algorithm for the detection of breast cancer. WOA based feature selection is done to extract optimal features from the dataset and to eliminate any unnecessary details. This is given as input to the ERT classifier and other algorithms. DT, KNN, SGD, RF, LR, KSVM, GNB and SVM are also used for classification and performance is compared with *BCD-WERT*. The results showed that *BCD-WERT* achieved the highest accuracy rate of 99.03% by using the WOA and ERT classifier. In the future, we intend to develop a software application to enable user predict if the cancer is benign or malicious. This application would benefit the society and will help the medical community to detect cancer at early stages. Machine learning and deep learning algorithms can be applied to the dataset to achieve better results and can be used in other areas of medical discipline.

## Supplemental Information

10.7717/peerj-cs.390/supp-1 Supplemental Information 1 Dataset and code for Breast Cancer Detection.

## Additional Information and Declarations

### Data Availability

The following information was supplied regarding data availability: Data is available at GitHub: https://github.com/Abdul-Rehman-J/BCDWERT.
